# Effects of properties of metal-contaminated soils on bacterial bioluminescence activity, seed germination, and root and shoot growth

**DOI:** 10.1186/s40064-016-1774-8

**Published:** 2016-03-03

**Authors:** Il-Mo Kang, In Chul Kong

**Affiliations:** Mineral Resources Department, Korea Institute of Geoscience and Mineral Resources, Daejeon, 34132 Korea; Department of Environmental Engineering, Yeungnam University, Kyungbuk, 38541 Korea

**Keywords:** Bioassay, Bioluminescence, Seed germination, Root/shoot growth, Soil extractants

## Abstract

This study examined the effects of several factors (metal contents and soil properties) on bacterial bioluminescence activity, seed germination and root/shoot growth of *Lactuca* in metal-contaminated soils. Each bioassay showed different sensitivities to extractants of soil samples. Average sensitivities of the bioassay were in the following order: root growth > bioluminescence ≥ shoot growth ≥ seed germination. Both total and weak acid-extracted metal contents showed no observable correlations with the activity of any bioassays (r^2^ < 0.279). However, reasonable correlations were observed between the bioluminescence activity and organics (r^2^ = 0.7198) as well as between root growth and CEC (r^2^ = 0.6676). Effects of soils were difficult to generalize since they were dependent on many factors, such as soil properties, metal contents, and the organism used in each test. Nonetheless, these results indicated that a battery of bioassays is an effective strategy for assessment of contaminated soils. Furthermore, specific soil factors were shown to more influence on soil toxicity, depending on the type of bioassay.

## Background

Metals are common contaminants in many parts of the environment and are responsible for numerous environmental and health problems (Shivhare and Sharma 2012). Metals are one of the most resistant contaminants affecting ecosystems due to their non-biodegradability and possible toxicity. For example, metalloid arsenic is one of the most toxic contaminants found in soil, which is produced by many industrial activities (Yi et al. 2007; Ravenscroft et al. 2009). Generally, soil pollution by metals is evaluated by chemical analysis of the concentrations of metals (Agnieszka et al. 2014). Soil monitoring in Korea is also based mainly on the maximum permissible chemical contents of two standards: concern standard and countermeasure standard. However, chemical data alone such as concentrations of contaminants are not sufficient to assess toxic effects on the ecosystem, as they do not provide information on the effects of contaminants on biota. Therefore, to assess risk of contaminated environments, chemical methods should complement existing biological and toxicological methods (Molnar et al. 2005; Leitgib et al. 2007).

Toxicity evaluation of contaminated environments has gained widespread attention over the past two decades (Banks and Schultz 2005). Toxicity bioassays provide information on the detection of contaminated chemicals as well as the bioavailability of substances that may harm the environment (Alvarenga et al. 2008). However, no individual bioassay can provide a true estimation of chemical toxicity since none has uniform sensitivity to all pollutants. Therefore, evaluation based on individual toxicity levels is of limited benefit unless it can be correlated with other data. Plaza et al. (2010) reported that it is essential to select appropriate organisms to test for different taxonomic groups and candidates representing different links of the trophic chain because organisms show variable sensitivity to different substances. To test chemical toxicity, various organisms such as bacteria, algae, protozoa, plants, and fish are utilized (US EPA 1993). In addition to whole organisms, several key metabolic processes of organisms, specifically enzyme activity or biosynthesis, and bacterial bioluminescence, have been used to assess toxicity of environmental systems. Plant processes have been performed for several different types of toxicity studies involving in environmental biomonitoring (Di Salvatore et al. 2008). For example, seed germination primarily assess acute toxic effects in the short-term, whereas long-term studies for both acute and chronic toxicities designed to evaluate plant biomass and root/shoot elongation over a 2–8 week period (Wang and Liu 2001). These plant bioassays are particularly proper for soil contamination (Boutin et al. 2004). Bacterial bioluminescence assay is also a time-saving, cost-effective test that is widely used for determining the acute toxicities of various type of samples (Wang et al. 2002).

The majority of toxicological studies have mainly focused on the toxicity of single or binary mixture chemicals under controlled conditions (Pavlaki et al. 2011). In practice, the environment is contaminated to various mixtures of chemicals. As such, studies on environmental samples may better reflect pollution of ecosystems and thus could assist in identifying ecologically relevant criteria. Unfortunately, determining the responses of organisms exposed to more than one chemical is one of the most difficult tasks in environmental risk assessment (Norwood et al. 2003). In addition to biotic responses to contaminants, abiotic characteristics of soil can strongly affect the bioavailability and toxicity of contaminants in soil. Many chemical and biochemical processes such as precipitation–dissolution, adsorption–desorption, and oxidation–reduction are critical processes in the control of bioavailability and mobility of metals in soil. The mobility of metals depends not only on their total concentrations in soil but also on soil properties [e.g., soil pH, particle size, organic matter content, redox potential, cation exchange capacity (CEC)], metal properties, and environmental factors (He et al. 2005). Metals present in various forms as water-soluble, exchangeable, oxide-bound, carbonate-bound, organic matter-bound, residual bound to resistant minerals (silicates), display variable mobility and bioavailability (Han et al. 2000). Water-soluble and exchangeable fractions are readily released into the environment, whereas residual fractions are immobile under natural conditions. Several studies have addressed the effects of soil factors on Cu and Ni toxicity on plant growth (Rooney et al. 2006; Li et al. 2013). For example, Warne et al. (2008) reported that soil CEC and pH are important soil factors for predicting Cu phytotoxicity across soils. Since metals are important global environmental contaminants, it is important to assess their effects by appropriate bioassays examining their soil properties for any risk assessment.

The purpose of this investigation was as follows: (1) to evaluate the toxicity of soils using different bioassays, (2) to examine possible relationships between the observed toxicity and metal contents (total and acid-extracted) or soil properties. The observed information may improve our understanding for risk assessment of metal-contaminated sites.

## Results

### Characteristics of metal contents of tested soils

Total and 0.1 N HCl acid-extracted metal concentrations of tested soils were presented in Table 1. Results showed that six metal concentrations in soil were approximately in the ranges of 127–920 and 1.03–23.02 mg/kg soil for total and acid-extracted fractions, respectively. More specifically, metal concentrations of soils were in the following ranges for total (1–627 mg As, 41–117 mg Zn, 40–265 mg Cr, 32–59 mg Pb, 12–95 mg Cu, and 1 mg Cd per kg dry soil) and acid-extracted (0.13–15.72 mg As, 0.5–6.51 mg Zn, 0.19–6.58 mg Cr, 0–4.60 mg Pb, 0.17–2.45 mg Cu, and 0.05–0.16 mg Cd per kg dry soil) fractions. Among the 11 samples, four (#A, B, C, D, K) were highly contaminated with high levels of arsenic within the range of 381–627 mg As/kg dry soil. Percentage distributions of the acid-extracted metal fraction were in the range of min. 0.3 % to max. 6.3 % (avg. 1.4 %) of total contents, depending on the sample site. Low correlation (r^2^ = 0.1864) was observed between contents of total and acid-extracted metals.

**Table 1:** Total and weak acid-extracted metal concentrations of tested soil samples

Samples	Metals (mg/kg dry soil) (total/acid-extracted)						
	As	Cd	Cu	Pb	Zn	Cr	Sum
#A	627/15.72^a^	1/0.14	58/1.90	55/0.18	113/2.57	66/0.42	920/20.9 (2.3 %)^b^
#B	566/0.32	1/0.09	45/1.71	52/2.63	99/1.44	59/0.98	822/7.2 (0.9 %)
#C	381/13.31	1/0.16	32/2.08	45/1.78	79/4.05	54/1.68	592/23.0 (3.9 %)
#D	433/0.16	1/0.05	83/0.60	48/1.95	117/0.63	57/0.69	739/4.1 (0.6 %)
#E	169/0.05	1/0.05	54/0.17	36/nd	100/0.62	56/0.19	416/1.1 (0.3 %)
#F	1/0.16	1/0.06	20/1.07	37/4.60	92/6.51	65/1.25	216/13.6 (6.3 %)
#G	1/0.21	1/0.06	18/0.33	44/1.31	98/1.01	45/0.20	207/3.1 (1.5 %)
#H	1/0.16	1/0.05	95/0.45	42/3.81	77/0.67	106/1.18	322/6.3 (2.0 %)
#I	1/0.13	1/0.06	12/0.20	32/0.72	41/0.94	40/0.27	127/2.3 (1.8 %)
#J	1/0.13	1/0.05	13/0.17	44/1.34	60/0.5	102/0.36	221/2.5 (1.2 %)
#K	401/0.32	1/0.10	47/2.45	59/0.62	54/2.61	265/1.25	827/7.4 (0.9 %)

^a^Total and acid-extracted concentrations of each metal

^b^Concentration of six metals: total/acid-extracted/ % of acid-extracted to total contents

### Effects of soils on bioluminescence activity

**Fig. 1:**
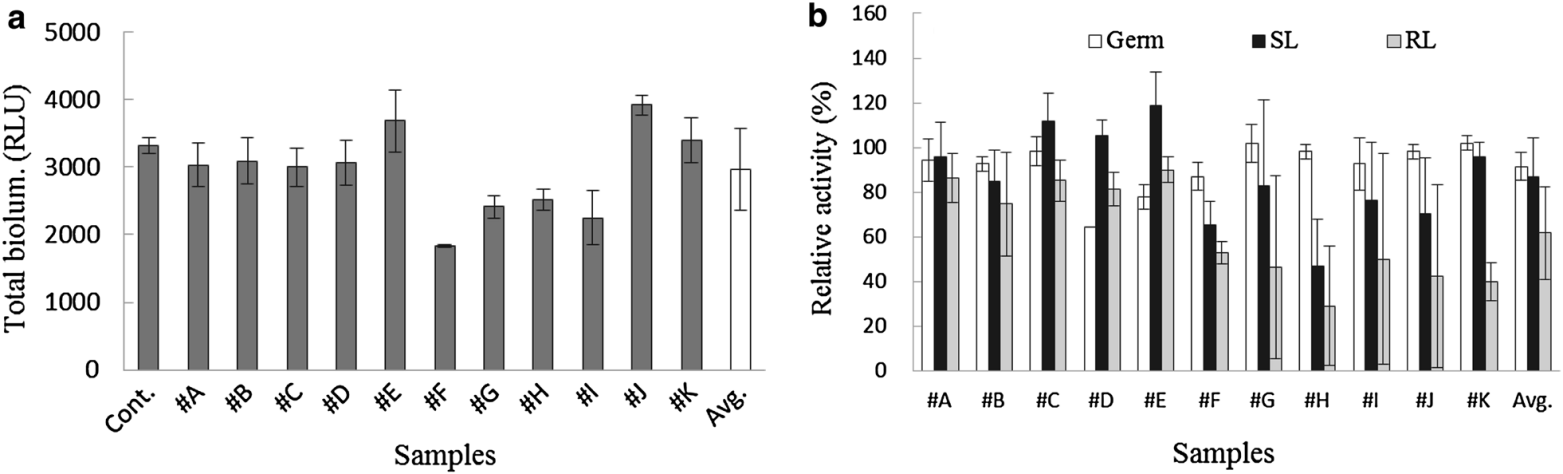
Comparisons of **a** total bioluminescence activities and **b** relative activities of germination, root, and shoot length to the control upon exposure to soil extractants

**Fig. 2:**
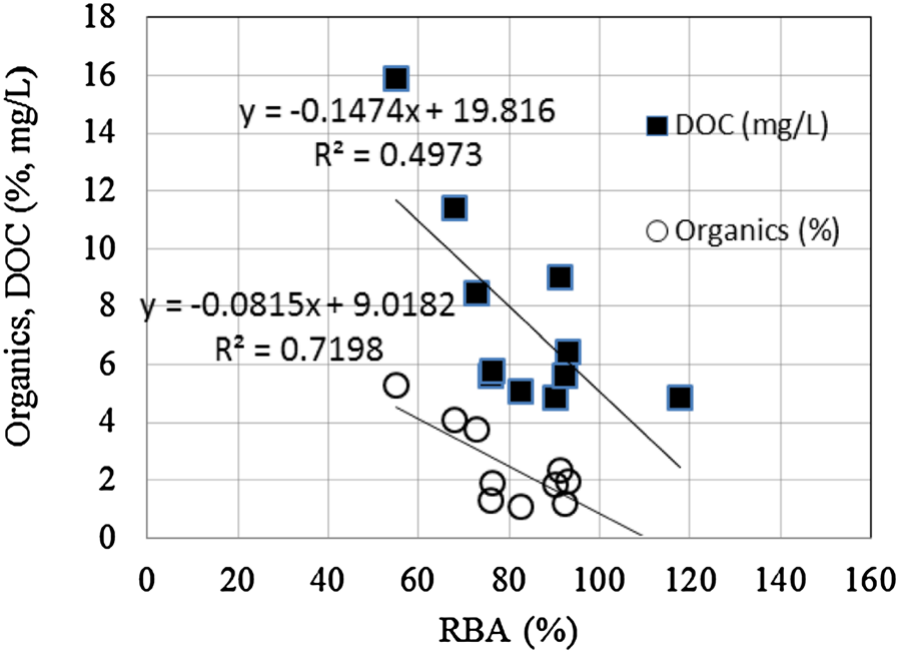
Correlations of organic contents and DOC with relative bioluminescence activity (RBA)

Figure 1a illustrated the effects of soil extractants in the tested bioassays based on total bioluminescence activity. Control (no exposure to soil) produced a total bioluminescence of approximately 3500 ± 116 RLU during 1.5 h of incubation. Bioluminescence activity was in the range of min. 55 % (1832 ± 55 RLU) to max. 118 % (3916 ± 155 RLU) of control during 1.5 h of exposure, depending on soil type. In general, sudden changes in bioluminescence activity were observed after exposure to extractants. For example, #B and #D samples showed elevated bioluminescence activities from 81 to 107 and 65 to 111 % of control after 1.5 h of exposure, respectively. In contrast, #A and #G samples showed inhibited bioluminescence activity from 115 to 75 and 91 to 51 % of control, respectively. Correlations between effects of soil extractants on bioluminescence activity and soil characteristics [metal contents (total and acid-extracted) or physicochemical characteristics (CEC, pH, DOC, and organics)] were examined. No considerable correlations (r^2^ values <0.1025) were observed between metal contents (total and acid-extracted) and effects on bioluminescence activity. However, reasonable correlations were observed with DOC and organics, which showed r^2^ values of 0.4973 and 0.7198, respectively (Fig. 2). Other soil properties such as pH and CEC showed no considerable correlations with bioluminescence activity (r^2^ < 0.096).

### Effects of soils on seed germination

In the control (no sample treatment), an average of 18 ± 1.7 seeds per batch of 20 seeds was germinated (greater than 2-cm growth) during the 3-day incubation period. Unlike the effects on bioluminescence activity (55–118 % of control), the percentage of seeds germinated in the presence of samples was in the range of 64–102 % of control (average 84 ± 26.6 %) (Fig. 1b). Correlations between effects of soil extractants on seed germination and soil properties were also examined. Overall, no significant correlations between seed germination by soil extractants and soil properties (CEC, pH, DOC, and organics) were observed. All cases showed no considerable correlations, with r^2^ values in the range of 10^−6^ (DOC) to 0.0052 (total metal contents).

### Effects of soils on root and shoot growth

Effects on relative root length (RRL) and relative shoot length (RSL) of *Lactuca* were measured in the metal-contaminated soils. Root and shoot lengths of the control ranged from 44–46 and 31–33 mm, respectively, depending on the batch. Of the tested soils, RSL and RRL were in the ranges of 47–119 and 29–90 %, respectively (Fig. 1b). RSL and RRL of the #H soil sample were 47 and 29 % of control, respectively, which represents the most prominent reduction. On the other hand in the #E sample, RSL and RRL were 119 and 90 % of control, respectively, which represents the least effect. Average effects on RSL and RRL were 85 ± 21.4 and 60 ± 22.2 %, respectively. Results of the correlation coefficient are presented on Table 2. Low correlations (less than approximately r^2^ value 0.27 for both) were observed between total metal contents and their effects on RRL and RSL, and correlations were even lower with acid-extracted contents (r^2^ value 0.169 for RRL, 0.0404 for RSL). However, interestingly, RRL was positively correlated with CEC, showing an r^2^ value of 0.6676. RSL also showed reasonable correlation with CEC compared to other properties with an r^2^ value of 0.3288, although it was slightly lower than that for RRL. Neither RRL nor RSL showed significant correlations with pH, DOC, and organics, showing r^2^ values ranging from 0.0182 to 0.1479. A positive correlation between RRL and RSL (r^2^ = 0.6849) was observed for the tested soil samples containing a wide concentration range for all metals.

**Table 2:** Summary of correlation coefficients between the activity of each bioassay and soil property

Parameters	Correlation coefficients (r^2^)						
	Total contents	Acid extracted	CEC	pH	DOC	Organic-C	Organics
Bioluminescence	0.103	<0.001	0.095	0.096	0.497	0.720	0.720
Seed germination	0.022	0.052	0.031	0.048	e−06	0.011	0.011
Root growth	0.279	0.169	0.668	0.086	0.028	0.018	0.018
Shoot growth	0.272	0.040	0.329	0.121	0.150	0.054	0.054

### Effect of extraction ratios (soil:water) on toxicity

**Fig. 3:**
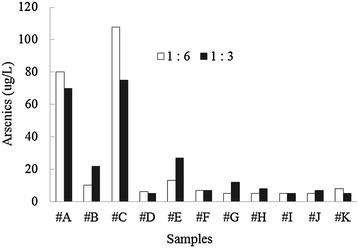
Comparison of arsenic concentration in water extractants at 1:6 and 1:3 (soil:water) ratios

Effects of extractants obtained at different ratios of soil to water (1:6 and 1:3) were compared based on the results of tested bioassays. Metal concentrations of water-extracted solution were very low (μg/L level), and no considerable differences were found between the two conditions (1:6 and 1:3). Total concentrations of all six metals showed lower than 500 μg/L (data not shown) in the water extractants. In the case of arsenic, 1:6 and 1:3 ratios resulted in concentrations of 75 and 108 μg/L for sample #C (total 381 mg/kg dry soil) as well as 70 and 80 μg/L for sample #A (total 627 mg/kg dry soil), respectively (Fig. 3). Total arsenic contents of all samples were 252 and 243 μg/L at 1:6 and 1:3 ratio, respectively. The average results of each bioassay, and correlations and statistical significances between the two results, are shown in Table 3. No statistical significant differences between the two results were observed in cases of bioluminescence activity, seed germination, and root growth (*p* value >0.1720). However, only statistical significant difference (*p* value 0.0191) was observed for shoot growth, which showed 105 ± 37.3 and 87 ± 21.4 % of RSL for 1:3 and 1:6 ratios, respectively. Results of two conditions showed a correlation in the range of 0.4587 to 0.6769. All relative activities to control were above 83 %, although low activities were observed for root growth (62 and 58 % at 1:6 and 1:3 ratios, respectively).

**Table 3:** Comparisons of toxicities, correlations, and statistical significances between two conditions (soil to water ratio 1:3 and 1:6)

Methods	Average activity (%) (soil to water ratio)		Correlation (r^2^) between two ratios	Statistics (*p* value)
	1:3	1:6		
Bioluminescence	92 ± 33.6	83 ± 16.5	0.4587	0.1720
Seed germination	95 ± 13.8	92 ± 11.4	0.6646	0.3393
Root growth	58 ± 23.0	62 ± 22.1	0.6246	0.4739
Shoot growth	105 ± 37.3	87 ± 21.4	0.6769	0.0191

## Discussion

Metals such as Cu, Cd, Cr, Pb, As, and Zn are all potential soil pollutants, and a wide range of these elements enters the environment through many sources, including industrial processes, mining, and irrigation (Liu et al. 2007). Tested soils, containing various ranges of metals, collected from nearby groundwater well collection sites. Leaching activities from these sites may have significance effects on groundwater quality and subsurface environments. Total metal contents of tested soils, excluding arsenic, were in the following order: Zn, Cr > Pb, Cu ≫ Cd. Arsenic content was in the range of 1–627 mg/kg soil and was highly variable depending on the site (Table 1). These metal concentrations were often higher than maximum permissible contents based on two standards in Korea (Jung 2001). As generally known by other investigators, cadmium displayed the lowest content.

Metal contents by weak acid (0.1 N HCl) extraction were in the range of 0.3–6.3 % of total contents (average 1.4 %), depending on the site. A low correlation (r^2^ = 0.186) between total and acid-extracted contents was observed. High percentage of Cd was found in the acid extractants, showing an average of 7.9 %. However, total concentrations of Cd in these soils were very low (~1.0 mg/kg soil), which means the corresponding absolute Cd concentrations in acid extractants were also very low (average 0.87 mg/kg soil). Percentage of acid extractants may vary according to soil characteristics, such as pH, organic contents, redox potential, as well as metal properties. Sample #F, which showed the highest percentage (6.3 %) of acid extraction, contained high contents of organics and DOC (5.31 % and 15.87 mg/L, respectively), whereas sample #E showing the lowest percentage (0.3 %) of acid extraction contained a low content of organics and DOC (1.09 % and 5.08 mg/L, respectively) compared to other samples. Correlation of acid extractant contents (% of total content) with organics (%), organic-C (%), and DOC showed r^2^ values of 0.3972, 0.3971, and 0.4866, respectively, whereas there were no observable correlations with soil properties such as pH and CEC, showing r^2^ values of 0.2084 and 0.0021, respectively. These results may be attributed to extraction of organic complex fraction metals by 0.1 N HCl. The distribution of fractions is an important parameter for determination of metal availability in soils. The organic fraction released in the oxidizable fraction is not considered to be very mobile or available since it is associated with stable high molecular weight humic substances, which slowly release small amounts of metals (Ure and Davidson 2001). Many research studies have also suggested that soil reactions, organic matter contents, and composition of the finest fraction may influence the mobility of metals in the environment (Venditti et al. 2000; Agnieszka et al. 2014). In this study, metal contents of water extractants, which close to exchangeable fraction in nature, were measured prior to the bioassays. Total metal contents of water extractants were very low, sometimes lower than the instrument detection limit, and less than 0.5 mg/L (<0.05 % of total contents) for all tested soils regardless of the soil to water ratio.

Kungolos et al. (2009) reported that the toxicity of single compounds varied up to two orders of magnitude, depending on the bioassay examined. Therefore, the combined results of different bioassays will better reflect effects in contaminated soils. In this study, various patterns appeared depending on the types of samples and bioassays (bacterial bioluminescence, seed germination, root, and shoot growth). In the case of bioluminescence, either inhibition or stimulation was observed with no complete inhibition during exposure periods. Relative bioluminescence activity (%) was in the range of max. 118 % and min 55 % of control (average 82 ± 16.4 %). Bioluminescence activity was considerably correlated with organic-C (%) (r^2^ = 0.7204) rather than with total and acid-extracted metal contents. For example, total metal contents of the #A and #F samples were 920 and 216 mg/kg soil, which were 91 % (9 % toxicity) and 55 % (45 % toxicity) of relative bioluminescence activity, respectively. This correlation might be attributable to the effects of organics themselves or the bioavailability of organic-metal complexes.

Liu et al. (2005) reported that seed germination is one of the best known indicators of plant development among other endpoints, including root length, shoot height, root biomass, shoot biomass, and total biomass. In contrast, Kapustka et al. (1995) reported germination as the least effective technique for vegetative response endpoints. In this study, seed germination was less sensitive than both root and shoot growth. Inhibition of specific enzymatic reactions by metals permeated into seed reserves is one of the main mechanisms behind metal toxicity on seed germination. In addition, seed germination activities of samples did not show any observable correlation with soil properties or metal contents, as all correlation coefficients were less than 0.0607. Effects on root and shoot growth, especially root growth, were clearly greater than that on seed germination (Table 3). Average root growth (62 ± 22.1 %) was nearly 0.75 to 0.67 times lower than bioluminescence activity, seed germination, and shoot growth (83 ± 16.5, 92 ± 11.4, and 87 ± 21.4 %, respectively). In general, an increase in metal concentration leads to reduction of root and shoot growth in plants. Therefore, root and shoot growth of germinated seeds are likely more affected by metals than seed germination itself. Differences between root and shoot toxicity might be due to the movement of metals from roots to shoots as well as direct contact with the root surface. As metals tend to be retained in root tissues, the effects are generally greater in roots than in shoots (An et al. 2004). Liu et al. (2007) also reported that plant measures are inhibited in the following order: root length > shoot height > biomass > germination frequency. Similar to reports, root growth was clearly more sensitive than shoot growth in all samples (An et al. 2004; Liu et al. 2005). RRL and RSL showed a positive correlation (r^2^ = 0.6849) regardless of the sample characteristics, indicating a reduction in the shoot growth strongly depended on the reduction of root growth.

Research has demonstrated that the toxicity and bioavailability of metals in soils can be strongly influenced by variations in soil chemical and physical properties (Langdon et al. 2014). The specific soil properties that have been shown to play the greatest roles in controlling toxicity, bioavailability, partitioning, and speciation of metals include pH, clay content, organic carbon, and CEC (Smolders et al. 2004; Oorts et al. 2006; Rooney et al. 2007; Criel et al. 2008; Heemsbergen et al. 2009; Li et al. 2011). In this investigation, no considerable correlation was observed between metal contents (total and acid extractants) and any results of the tested methods, showing all r^2^ values less than 0.2788. However, specific soil properties were considerably correlated with certain bioassays. Bioluminescence activity was highly correlated with organic-C (%), showing a correlation coefficient of r^2^ 0.7204. In contrast, root growth was highly correlated with CEC, showing a correlation coefficient of r^2^ = 0.6676, whereas correlations with other soil properties were in the range of 0.0183–0.2788. Shoot growth also showed a little high correlation (r^2^ = 0.3288) with CEC. Both RSL and RRL showed a better correlation with total metal contents (r^2^ = 0.2723, 0.2788) compared to other observations, showing r^2^ values of 0.1025 for bioluminescence and 0.0104 for seed germination. The effects of these soil properties on the behavior and availability of metals have been shown to be metal-specific, with different properties, or combinations of properties, having the greatest influence. Due to the complex characteristics of soil, it is difficult to generalize any relationship with toxicity. Li et al. (2013) reported that soil pH and organic-C are the most important soil properties controlling the effects of Cu and Ni toxicity on tomato and Bokchoy shoot growth. Other studies reported that one metal species in a mixture can influence or even decrease uptake of other metals, which may constitute a novel reduction mechanism (Peralta-Videa et al. 2002). Therefore, it seems not possible to apply the relationships between toxicity and soil properties that have been observed for one case to the behavior and availability of other cases.

## Conclusion

In conclusion, no considerable relationships were observed between toxicity and metal contents (total and acid-extracted). However, soil organic contents and CEC were found to be the main soil properties responsible for effects on bioluminescence activity and root growth, respectively. These factors might be directly related to the toxic effects or indirectly by increasing the bioavailability of contaminants. This result also indicated that root growth was a more sensitive measure of toxicity of contaminated soil, compared to other tested methods (bacterial bioluminescence activity, seed germination, and shoot growth). Overall, the effects of soils were difficult to generalize since they were dependent on many factors, such as metal contents, metal types, soil properties, and the organism used in each bioassay. However, this result suggested that, due to the different sensitivity of each bioassay, a battery of bioassays as opposed to just a single assay was a better strategy for assessment of environmental samples. In addition, observable correlations between specific toxicity and soil properties could be used as valuable information for soil assessments and prediction of toxicity in soils with a wide range of physicochemical properties.

## Methods

### Soil collection and metal contents

Soil samples were collected from 11 different sites in Korea near groundwater collection wells for drinking water. Five samples from each site were collected, and the mixture was used as a test soil sample. In the laboratory, soil samples were dried at ambient temperature (22–25 °C), crushed in a porcelain mortar, and sieved through a 2-mm screen. The air-dried samples were then stored in cloth bags for subsequent analysis. General soil properties are shown in Table 4. Total and 0.1 N HCl acid-extracted metal (Cu, Cd, Cr, Pb, Zn, and As) contents of soils were quantified by using Flame Atomic Absorption Spectrometry (AAS, Shimadzu, Japan) or Inductively Coupled Plasma (Perkin Elmer, USA). For toxicity test, each sample mixed with water (soil to water 1:6 and 1:3 ratios) was shaken for 6 h, after which the supernatant was used. The toxicity was studied using four bioassays: bacterial bioluminescence activity, seed germination, root growth, and shoot growth.

**Table 4:** Physicochemical characteristics of tested environmental soils

Samples	CEC (cmol/kg)	pH	DOC (mg/L)	Organic-C (%)	Organics (%)
#A	22.71	6.01	9.02	1.37	2.36
#B	14.99	5.70	6.44	1.12	1.94
#C	17.26	5.83	4.85	1.08	1.86
#D	11.69	5.82	5.61	0.71	1.22
#E	13.56	8.47	5.08	0.63	1.09
#F	8.02	5.00	15.87	3.08	5.31
#G	5.11	5.52	8.45	2.20	3.79
#H	7.31	5.81	5.60	0.75	1.29
#I	3.62	5.02	11.43	2.37	4.08
#J	5.52	7.73	4.88	−0.18	−0.30
#K	8.21	5.49	5.81	1.11	1.92

### Toxicity with bioluminescence bioassay

Ecotoxicity of soil extractants were determined using a bioluminescent mutant strain, *Escherichia coli* DH5 RB1436. This mutant contains a spontaneously deleted pUCD615 plasmid, which results in translocation of a constitutive promoter in the plasmid into close proximity to the lux-genes (Ko et al. 2012). This *E. coli* strain (obtained from Dr. R.S. Burlage of the University of Concordia, USA) has the ability to release bioluminescence during its growth phase. Strains were stored at −70 °C until needed, at which time they were grown overnight in Luria-Bertanika (LB^ka^) medium (tryptone 10 g, yeast extract 5 g, NaCl 5 g, 2 N NaOH 0.5 mL, kanamycin 50 mg/L) at 27 °C with shaking (130 rpm). The strains were then diluted to 1:30 in LB^ka^ medium and allowed to grow until the optical density (OD_600_) was approximately 0.6. This culture was appropriately diluted with minimum salt medium (MgSO_4_·7H_2_O 0.2 g, CaCl_2_ 0.1 g, FeSO_4_·7H_2_O 0.05 mg, NaMoO_4_·2H_2_O 0.05 mg, K_2_HPO_4_ 0.43 g, KH_2_PO_4_ 0.23 g), and the final optical density for the toxicity test was OD_600_ = 0.2. For the bioassay, 1 mL of the bacterial strain was mixed with 9 mL of soil extractants and incubated for 1.5 h. Bioluminescence was measured using a Turner 20/20 luminometer (Turner Design Inc., CA), where the maximum detection limit was 9999 relative light units (RLU).

### Toxicity with seed germination assay

Seeds of lettuce (*Lactuca sativa* L.) were selected based on its importance as a food crop. Prior to the germination test, all seeds were surface-sterilized in 3 % H_2_O_2_ and then rinsed with distilled water. Filter paper was placed on a Petri dish and moistened with 5 mL of the soil extractants. Controls were maintained by moistening the filter paper with 5 mL of distilled water. Twenty seeds of each species were then placed on a dish, which was covered by a lid and incubated in the dark at 23 ± 2 °C. Germinated seeds were counted after 3 days of incubation. When both the plumule and radical extended longer than 2 cm from their junctions, germination was confirmed. Triplicate sets were performed for each treatment.

### Toxicity with root and shoot growth bioassay

Germinated seeds were transferred to serum vials (five germinated seeds per vial) containing 50 mL of soil extractant solution. The vials were then placed in plant incubator at 25 °C for 4 days. Following incubation, shoot and root lengths were measured from the root–shoot junction to the longest tip. Root and shoot lengths of seedlings grown in test solutions were expressed as the percentage inhibition (%) of RRL and RSL compared with the control.

### Statistical analysis

Each of the experimental values was compared to its corresponding control. Statistical significance between different samples was accepted when the probability of the result assuming the null hypothesis (*p*) was less than 0.05. Statistical analysis of experimental groups utilized Student’s *t* test (http://www.graphpad.com).
